# Bioactivity Assessment and In Silico Target Engagement of *Clerodendrum infortunatum* Extracts: Correlating Experimental Outcomes With DFT and Docking Predictions

**DOI:** 10.1002/fsn3.72047

**Published:** 2026-07-01

**Authors:** Mohi Uddin, Sabikun Naher, Md. Hossain Rasel, Md. Jahirul Islam Mamun, Md. Nazmul Hasan, Nazmul Hasan Eshaque, Md. Mahmudul Hasan

**Affiliations:** ^1^ Department of Pharmacy, Faculty of Biological Sciences University of Chittagong Chittagong Bangladesh; ^2^ Department of Chemistry and Biochemistry University of Wisconsin‐Milwaukee Milwaukee Wisconsin USA

**Keywords:** ADME/T, analgesic, antidiarrheal, anti‐inflammatory, antipyretic, *Clerodendrum infortunatum*, DFT, molecular docking

## Abstract

*Clerodendrum infortunatum* (Lamiaceae) is commonly employed in traditional healing practices to manage inflammation, fever, pain, and gastrointestinal ailments. Despite its widespread use, there is limited scientific evidence supporting these therapeutic claims and insufficient characterization of its active phytochemicals. This study aimed to investigate the anti‐inflammatory, analgesic, antipyretic, and antidiarrheal properties of the methanolic extract (MECI) and n‐hexane fraction (NHF) derived from *C. infortunatum* leaves through integrated in vivo and *in silico* methodologies. Anti‐inflammatory activity was evaluated using protein denaturation and carrageenan‐induced paw edema models; analgesic effects were assessed via acetic acid‐induced writhing and tail immersion tests; antipyretic and antidiarrheal activities were examined using yeast‐induced pyrexia and castor oil‐induced diarrhea models, respectively. Additionally, molecular docking, ADME/T (absorption, distribution, metabolism, excretion/toxicity), DFT, and PASS (Prediction of Activity Spectra for Substances) analyzes were performed to explore the pharmacokinetic profiles and potential molecular targets of the identified compounds. MECI demonstrated strong inhibition of protein denaturation (89.29%) with an IC₅₀ of 44.10 μg/mL, comparable to diclofenac sodium. Both MECI and NHF significantly attenuated carrageenan‐induced paw edema and writhing responses in a dose‐dependent manner (*p* < 0.001), increased latency in the tail immersion test, and effectively reduced both yeast‐induced fever and castor oil‐induced diarrhea. In silico docking revealed that hydrocinnamic acid, o‐[(1,2,3,4‐tetrahydro‐2‐naphthyl)methyl]‐, exhibited high binding affinities for COX‐2 (−9.1) and the M_3_ muscarinic receptor (−10.2 kcal/mol), while ethyl isoallocholate showed notable affinity for mPGES‐1 (−5.6 kcal/mol). ADME/T, DFT, and PASS predictions indicated favorable oral bioavailability and low toxicity for the major constituents. The findings substantiate the traditional medicinal uses of *C. infortunatum*, demonstrating its multi‐target pharmacological actions mediated through COX‐2, mPGES‐1, and M3 muscarinic receptor pathways. Hydrocinnamic acid derivatives and ethyl isoallocholate emerge as promising candidates for the development of new anti‐inflammatory, analgesic, and antipyretic therapeutics.

## Graphical abstract

*Clerodendrum infortunatum* (Lamiaceae) is widely used in traditional medicine across South and Southeast Asia for the treatment of inflammation, fever, pain, and gastrointestinal disorders, yet its pharmacological efficacy and phytochemical basis remain inadequately validated by scientific research. To address this gap, the present study employed an integrated in vivo and *in silico* approach to evaluate the anti‐inflammatory, analgesic, antipyretic, and antidiarrheal activities of both the methanolic extract (MECI) and the n‐hexane fraction (NHF) prepared from *C. infortunatum* leaves. By combining standardized animal models with molecular docking against relevant biological targets, this work aims to provide empirical support for its ethnopharmacological uses and identify potential bioactive constituents responsible for its therapeutic effects.

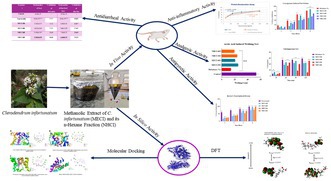

## 1 Introduction

Pain and inflammation are immune defense mechanisms activated by injury, irritants, or pathogens, leading to symptoms such as pain, redness, swelling, immobility, and heat. However, persistent or uncontrolled inflammation contributes to the development of various chronic disorders, including rheumatoid arthritis, atherosclerosis, and ischemic heart disease. Moreover, both infectious diseases (e.g., tuberculosis, leprosy, syphilis) and noninfectious conditions (e.g., asthma, peritonitis, inflammatory bowel disease, vasculitis, nephritis, celiac disease, and other autoimmune disorders) often manifest with symptoms of pain and inflammation (Chen et al. 2017; Wahid et al. 2021). Steroidal and nonsteroidal anti‐inflammatory drugs (NSAIDs) are commonly administered to alleviate the aforementioned symptoms; however, prolonged use of these medications may lead to adverse effects on various organs, including the kidneys, liver, gastrointestinal tract, cardiovascular system, central nervous system, and lungs (Bindu et al. 2020), (Reddy et al. 2025). Therefore, despite the wide availability of analgesic and anti‐inflammatory drugs, there remains a pressing need to develop new agents with improved efficacy and fewer side effects. Medicinal plants are among the most promising sources for such discoveries, as they are rich in bioactive phytochemicals. Pain and inflammation are commonly associated with a variety of conditions, including rheumatism, pneumonia, fibrosis, esophagitis, encephalitis, cancer, and cardiovascular diseases. Although nonsteroidal anti‐inflammatory drugs (NSAIDs) are frequently prescribed to manage inflammatory pain, their prolonged use can result in significant adverse effects. Consequently, numerous plants are investigated each year for their potential analgesic and anti‐inflammatory properties; however, only a limited number ultimately gain approval for clinical use following extensive and rigorous research (Sen et al. 2010), (Rasel et al. 2026).

Among life‐threatening diseases, diarrhea remains a major global public health concern, particularly in developing countries. In 2017, it accounted for approximately 8% of deaths among children under the age of five, making it the second leading cause of mortality in this age group. The condition is especially prevalent among young children, posing a significant threat to child health and survival (Hartman et al. 2023). Diarrhea can result from a wide range of bacterial, viral, and parasitic infections. In developed regions such as Europe and North America, viral infections—often showing a distinct winter seasonality—are responsible for the majority of diarrheal cases. In contrast, in developing countries with poor sanitation and limited hygiene practices, enteric parasitic infections are more prevalent and tend to peak during the summer months (Hossain et al. 2026), (Podewils et al. 2004). At present, the primary approach to managing diarrhea involves the use of agents that slow down gastrointestinal transit. Commonly used drugs in this category include loperamide, diphenoxylate, codeine, and diluted opium tincture (Nightingale 2022). Research findings suggest that liver abscesses frequently occur following episodes of gastroenteritis that have been treated with antidiarrheal medications (Thomas et al. 2017). In addition, commonly reported side effects of loperamide include constipation, vomiting, drowsiness, and dizziness (Vardanyan 2017).

The term “fever” can be used interchangeably. Fever is defined as a regulated increase in body temperature above the normal physiological range. The average normal oral body temperature is between 36.7°C and 37°C (98°F–98.6°F). Rectal measurements are typically about 0.6°C (1°F) higher, while axillary readings are approximately 0.6°C (1°F) lower than oral temperatures. Skin temperature is also generally lower than oral temperature, though it may vary depending on the measurement method used (DiPiro et al. 2014; Estella et al. 2022). Fever represents a complex, adaptive response of the body to diverse immune challenges, whether caused by infectious or noninfectious factors (Ogoina 2011), (Roy et al. 2024).

Researchers in biology are increasingly leveraging computational biology to create significant opportunities for validating their findings. In silico screening can effectively predict the pharmacological activities of phytochemicals. Computer‐aided drug design (CADD) techniques, including molecular docking, have proven valuable for studying and developing drug candidates within a relatively short timeframe. An effective molecular docking analysis should accurately determine the positioning of ligands at the binding site and elucidate the physicochemical interactions within the protein structure (Hossen et al. 2021; Mohammad et al. 2025).

*Clerodendrum infortunatum* Linn. (Verbenaceae; known as Ghentu in Bengali, Bhat in Hindi, and Bhania in Oriya) is a terrestrial shrub characterized by a square, dark‐colored stem and simple, opposite, decussate leaves. The leaves are petiolate, exstipulate, coriaceous, and hairy, and the plant emits a distinct, unpleasant odor (Verma et al. 2025; Kirtikar and Basu 1996). Different parts of *C. infortunatum* have been traditionally used by tribal communities to treat colic, scorpion stings, snake bites, tumors, and certain skin disorders. Additionally, it has been employed in folk medicine for managing bronchitis, asthma, fever, blood‐related ailments, inflammation, burning sensations, and epilepsy (Kumar et al. 2025; Modi et al. 2010). Phytochemical analyzes have shown that it possesses bioactive compounds, including triterpenes, steroids, and flavonoids (Ranatunge and Soysa 2024; Akihisa et al. 1989; Sinha et al. 1981). The plant's reported antioxidant, antimicrobial, anthelmintic, and analgesic properties have generated considerable interest, driving further research into its therapeutic potential (Changad et al. 2024).

Herbal remedies have long been explored for their therapeutic benefits due to their rich content of bioactive compounds and relatively low side effects. Among these, *Clerodendrum infortunatum* has gained attention for its wide range of pharmacological activities. The present study thoroughly evaluated the anti‐inflammatory, analgesic, antidiarrheal, and antipyretic activities of the methanolic extract of *Clerodendrum infortunatum* leaves (MECI) and its n‐hexane fraction (NHF) using established experimental models. Additionally, *in silico* molecular docking and ADMET analyzes were conducted to explore potential mechanisms of action and assess the pharmacokinetic and toxicological properties of the bioactive compounds, providing insights into their therapeutic potential.

## 2 Methods and Materials

### 2.1 Chemicals and Reagents

Diclofenac sodium (Square Pharmaceuticals Ltd., Bangladesh) was employed as the standard for analgesic and anti‐inflammatory tests, whereas loperamide and paracetamol were used as reference drugs for the antidiarrheal and antipyretic evaluations, respectively. Fractionation solvents, including n‐hexane and methanol, were procured from Merck at analytical grade. All other chemicals used in the study were also of analytical grade and required no additional purification.

### 2.2 Plant Materials Collection and Identification

For the current study, *Clerodendrum infortunatum* was collected from the Bandarban hill district of Bangladesh in August 2024. The plant specimen was subsequently authenticated by Dr. Shaikh Bokhtear Uddin, a taxonomist and professor in the Department of Botany.

### 2.3 Extraction and Partitioning Process

Plant materials were dried and ground into a fine powder (750 g) using a high‐performance electric grinder (brand name: Damai), then soaked in 4 L of methanol for 15 days at room temperature (23°C ± 0.5°C). Filtrate obtained by using cheesecloth and Whatman filter paper No. 1 was concentrated under reduced pressure at a temperature below 45°C using a rotary evaporator (RE200, Bibby Sterling, UK). The solvent was completely removed by using a rotary evaporator. The crude methanolic extract (MECI) obtained was 29 g, corresponding to a yield of 3.87% of the starting material, and was thereafter stored in airtight containers for further analysis (Ali et al. 2025). Crude extracts undergo solvent‐solvent partitioning using the technique developed by Kupchan and Tsou; a modified version by Wagenen et al. (Beckett and Stenlake 1986) using the solvent n‐hexane.

### 2.4 Experimental Animal and Ethical Statement

The study employed Swiss albino male mice aged 4–5 weeks, with body weights ranging from 25 to 35 g. The animals were sourced from the Animal House of Jahangirnagar University, Dhaka, and housed in clean, dry polypropylene cages under controlled environmental conditions. They were maintained on a 12‐h light/dark cycle at 25°C ± 2°C and 45%–55% relative humidity. Before experimentation, the mice were acclimatized to the laboratory environment for 3–4 days and provided with a standard laboratory diet and unrestricted access to water. To ensure uniform experimental conditions, food was withheld 12 h before and during the procedures. All experimental protocols followed the Guidelines for Scientific Experiments on Animals (Swiss Academies of Medical and Natural Sciences, 1995) and OECD guidelines (2001). Ethical approval was granted by the Animal Ethics Review Board (AERB), Faculty of Biological Sciences, University of Chittagong (Approval No: AERB‐FBSCU‐202405876‐(4)).

### 2.5 Experimental Design

To evaluate in vivo anti‐inflammatory, analgesic, antidiarrheal, and antipyretic activities, 20 experimental animals were randomly selected for each test and divided into four groups of five mice each. Group I served as the control and received 1% Tween‐80 and DMSO in saline (10 mL/kg, p.o.). Group II was administered the standard drugs—diclofenac sodium, loperamide, or paracetamol—depending on the specific activity being tested. Groups III and IV were given oral doses of 200 or 400 mg/kg, respectively, of either the methanolic extract or the n‐hexane fraction of the methanolic extract.

### 2.6 Anti‐Inflammatory Activity

#### 2.6.1 Egg Albumin Denaturation Assay

The assay was carried out as described by Sakat et al. (2010) with modifications. The reaction mixture (5 mL) consisted of 0.2 mL of egg albumin (from a fresh hen's egg), 2.8 mL of phosphate‐buffered saline (pH 6.4), and 2 mL of plant extracts at varying concentrations (15.625, 31.25, 62.5, 125, 250, and 500 μg/mL). Double‐distilled water of equal volume was used as the control. The mixtures were incubated at 37°C ± 2°C for 15 min, followed by heating at 70°C for 5 min. After cooling, absorbance was measured at 660 nm, with the vehicle serving as the blank. Diclofenac (1 mg/mL) was used as the reference drug and processed similarly for absorbance measurement. The percentage inhibition of protein denaturation was calculated using Equation (1):

(1) $$\%\text{Inhibition}=\frac{\left(A_{C}-A_{S}\right)}{A_{C}}\times 100$$

where *A*_*C*_ is the absorbance of the control group and A_S_ is the absorbance of the sample.

#### 2.6.2 Carrageenan‐Induced Paw Edema Test

Each group was administered the specific treatments outlined in the experimental design. Before treatment, each mouse was accurately weighed, and the doses of the test samples and control substances were adjusted based on their individual body weights (Nahar et al. 2025). Carrageenan was injected into the subplantar region of the mice's left hind paw, and paw volume was measured at 1, 2, 3, and 4 h following the method described by Nair et al. (2012). Paw edema was expressed as the change in paw circumference (cm) by using the following Equation (2):

(2) $$\text{Inhibition of edema}\;\left(\%\right)=\frac{\left(M_{t}-M_{0}\right)\text{control}-\left(M_{t}-M_{0}\right)\text{treated}}{\left(M_{t}-M_{0}\right)\text{control}}\times 100$$

Here, *M*_*t*_ = Mean paw circumference for each group at different time intervals, *M*_*0*_ = Mean paw circumference for each group before carrageenan injection.

### 2.7 Analgesic Activity

#### 2.7.1 Acetic Acid‐Induced Writhing Method

The acetic acid‐induced writhing test was conducted as previously described by Mamun‐Or‐Rashid et al. (Mamun‐Or‐Rashid et al. 2017). Each group was administered the specific treatments outlined in the experimental design. Thirty minutes after treatment, 0.7% acetic acid (10 mL/kg body weight) was injected intraperitoneally. Abdominal contractions, or writhes, were counted for each group from 5 to 20 min after the acetic acid injection, and the results were expressed as a percentage of protection. The percentage protection against acetic acid was calculated using the following equation (3):

(3) $$\%\text{of Inhibition}=\frac{\mathrm{Nc}-\mathrm{Nt}}{\mathrm{Nc}}\times 100$$

Here, *Nc* = number of writhings in control, and *Nt* = number of writhings in test animals.

#### 2.7.2 Tail Immersion Test

The central analgesic activity of the extracts was investigated in Swiss albino mice using the tail immersion test, following the method described by Kumar and Shankar (Kumar and Shankar 2009). Animals in each group received their respective treatments according to the experimental design. During the test, approximately 2–3 cm of the mouse tail was immersed in a water bath maintained at 50°C ± 1°C. The baseline tail withdrawal latency prior to treatment was determined as the average of three readings taken at 2‐min intervals. After administration of the treatments, the withdrawal latency was recorded at 30, 60, 90, and 120 min using the same procedure. Mice exhibiting baseline latency values outside the 3–5 s range were excluded from the experiment. To prevent thermal injury, a 15‐s cut‐off time was applied. The central analgesic activity was expressed as the percentage of maximal possible effect (%MPE).

### 2.8 Antidiarrheal Activity

#### 2.8.1 Castor Oil (CO)‐Induced Diarrhea Model

The study was conducted using a slightly modified version of the method described by Ferede et al. (2021). The experiment commenced with an 18‐h fast in the mice. Treatments were administered according to the guidelines outlined in Section 2.5 of the experimental design. 1 h after treatment, each mouse received 0.5 mL of castor oil by gavage. The animals were individually housed in cages lined with blotting paper, and the amounts of wet and dry feces were recorded hourly for 4 h, with fresh blotting paper provided at the start of each hour (Hasan, Momen, et al. 2025). The percentage inhibition of antidiarrheal activity was then calculated using the following Equation (4):

(4) $$\%\text{Inhibition}=\left\{\frac{\left(A-B\right)}{A}\right\}\times 100$$

where *A* = average number of diarrheal feces in the control group and *B* = average number of diarrheal feces in the treated group.

### 2.9 Antipyretic Activity

#### 2.9.1 Brewer's Yeast‐Induced Pyrexia Method

For this study, the methodology mentioned by Sahu et al. (2016) was applied. Rectal temperatures were recorded at predetermined intervals to monitor each Swiss albino male mouse's body temperature. Before the experiment, the mice were fasted overnight with free access to water. Pyrexia was induced by subcutaneously injecting the dorsum of each mouse with a 20% (w/v) brewer's yeast suspension (10 mL/kg). Rectal temperatures were re‐measured 24 h after yeast administration, and mice that did not exhibit a minimum temperature increase of 0.5°C within this period were excluded from the study (Mohammad et al. 2025). The twenty selected mice were treated according to the protocol outlined in the experimental design section. Thirty minutes after treatment, a digital thermometer (SK‐1250 MC, Sato Kiriaki Mfg. Co. Ltd., Japan) was gently inserted into the rectum of each mouse, and the rectal temperature was recorded. The percentage reduction in pyrexia was then calculated using the following Equation (5):

(5) $$\%\text{of reduction of pyrexia}=\frac{B-C}{B-A}\times 100$$

Here, *A* = Normal body temperature; *B* = Rectal temperature at 24 h after yeast administration; *C* = Rectal temperature after drug administration at a different time interval.

### 2.10 In Silico Study

#### 2.10.1 ADME and Drug‐Likeliness Prediction

The Absorption, Distribution, Metabolism, and Excretion (ADME) properties of the bioactive compounds from *C. infortunatum* were assessed using Lipinski's Rule of Five, along with computational tools SwissADME and pkCSM, to evaluate their drug‐likeness and pharmacokinetic profiles (Lipinski 2004; Mamun, Alim, et al. 2026).

#### 2.10.2 Ligand Preparation

Fifteen minor metabolites of *C. infortunatum* (Dey et al. 2015) were chosen as ligands for the docking studies. Their three‐dimensional (3D) SDF structures were obtained from the PubChem database. For those compounds without available 3D SDF files, two‐dimensional (2D) SDF structures were downloaded and subsequently converted to 3D format using Open Babel software (O'Boyle et al. 2011). Before docking simulations, all ligand structures were energy‐minimized and converted into the .pdbqt format using AutoDock Tools (version 1.5.6) to ensure proper preparation for computational docking (Hasan, Alim, et al. 2025).

#### 2.10.3 Protein Preparation

The crystal structures of the target proteins were obtained from the RCSB Protein Data Bank, including human cyclooxygenase‐2 receptor (PDB: 5IKR), M_3_ muscarinic acetylcholine receptor (PDB: 5ZHP), and microsomal prostaglandin E synthase‐1 (mPGES‐1) enzyme (PDB: 4YK5). COX‐2 (5IKR) was used for the evaluation of anti‐inflammatory and analgesic activities, M_3_ muscarinic receptor (5ZHP) for antidiarrheal activity, and mPGES‐1 (4YK5) for antipyretic activity. The active sites of these enzymes were identified based on previously published data by Kurumbail et al. (1996). Swiss‐PdbViewer (v4.1) and BIOVIA Discovery Studio 4.5 Client were employed to prepare the protein structures by removing water molecules, cofactors, and heteroatoms for proper processing. Hydrogen atoms were then added, and the protein structures were energy‐minimized using the MMFF94 force field within the PyRx virtual screening tool. To ensure compatibility and streamline the molecular docking studies, the processed protein structures were saved in PDB format throughout the docking procedure (Mamun, Mizan, et al. 2025).

#### 2.10.4 Molecular Docking Study

PyRx AutoDock Vina 1.2.0, featuring updated docking algorithms, an expanded force field, and Python integration, was used to dock selected proteins with *C. infortunatum* ligands (Mamun, Rasel, et al. 2026). The proteins were considered rigid, while the ligands were treated as semiflexible. Docking results with root mean square deviation (RMSD) values below 2 Å were considered acceptable. Active sites were identified based on the positions of cocrystallized ligands, which were redocked to validate the methodology. After validation, AutoGrid generated grid boxes with a spacing of 0.278 Å for three target proteins, tailored to their respective active sites: PDB ID 5IKR (X: 47.03, Y: 27.33, Z: 16.23 Å), PDB ID 5ZHP (X: 37.13, Y: 31.05, Z: 25.87 Å), and PDB ID 4YK5 (X: 41.07, Y: 31.65, Z: 10.64 Å). The grid box dimensions were selected to fully cover the active site without being excessively large, ensuring docking specificity. These dimensions were carefully selected to fully encompass the binding cavities of each receptor, ensuring comprehensive sampling of ligand–protein interactions during molecular docking and enabling accurate prediction of binding poses and affinities. Docking interactions were visualized and analyzed using BIOVIA Discovery Studio Visualizer 2020 (Dallakyan and Olson 2015; Mamun, Mizan, et al. 2026).

### 2.11 Ligand's Profiling and Optimisation

Quantum mechanical calculations were performed using density functional theory (DFT) with the B3LYP hybrid functional and the 6‐31G basis set to optimize molecular geometries and evaluate key electronic properties. Frontier molecular orbital (FMO) analysis—specifically the energies and spatial distributions of the highest occupied (HOMO) and lowest unoccupied (LUMO) molecular orbitals—was conducted to assess chemical reactivity and kinetic stability. The HOMO–LUMO energy gap (ΔE = E_LUMO_—E_HOMO_) served as a reactivity descriptor: a smaller ΔE correlates with higher reactivity (and lower kinetic stability), whereas a larger gap indicates greater inertness. Optimized low‐energy conformers, validated using virtual screening protocols—including tautomer enumeration, protonation‐state assessment, and bond‐order correction—were subsequently used for molecular docking and binding‐affinity prediction. To enhance computational reliability, geometry optimisations incorporated polarization functions (e.g., via the 6‐31G(d) basis) and, where applicable, dispersion‐corrected B3LYP (e.g., B3LYP‐D3(BJ)) to better model noncovalent interactions. Global reactivity descriptors, including electronegativity (χ), chemical hardness (η), softness (σ), and electrophilicity index (ω), were derived from FMO energies using conceptual DFT principles. Final optimized structures were converted to PDB format to enable seamless integration with structure‐based drug design pipelines, including molecular docking, dynamics simulations, and in silico ADMET profiling. This integrated quantum‐to‐classical modeling framework bridges electronic‐structure insights with pharmacological prediction, supporting rational lead optimisation (Ruan et al. 2017; Tumilaar et al. 2025).

$$\begin{array}{l} I=-\text{ELUMO}\\ A=-\text{EHOMO}\\ \left(Χ\right)=\left(I+A\right)/2\\ \omega =\mu 2/2\eta \\ \mu =\left(-I+A\right)/2\\ \eta =\left(I–A\right)/2\\ S=1/\eta \end{array}$$

Here, HOMO = Highest Occupied Molecular Orbital, LUMO = Lowest Unoccupied Molecular Orbital, A = Electron affinity, I = Ionization potential, X = Electronegativity, ω = Electrophilicity index, μ = Chemical potential, η = Chemical hardness.

### 2.12 PASS Prediction

The biological activities of *C. infortunatum* compounds were predicted using the PASS (Prediction of Activity Spectra for Substances) online server, which calculates the probability of activity (Pa) and probability of inactivity (Pi) for different pharmacological effects. The compounds, initially retrieved from PubChem, were converted into SMILES format for the computational analysis (Clements 2017). To explore their potential therapeutic effects, the compounds were analyzed using the terms “anti‐inflammatory”, “analgesic”, “antidiarrheal”, and “antipyretic”, allowing the prediction of Pa and Pi values for anti‐inflammatory, analgesic, antipyretic, and anti‐arthritic activities, respectively (Mamun, Rasel, et al. 2025).

### 2.13 Statistical Analysis

Results for each concentration are expressed as mean ± standard error mean (SEM) from triplicate measurements. Statistical analysis was performed using one‐way analysis of variance (ANOVA) followed by Dunnett's *t*‐test for post hoc comparisons, using GraphPad Prism software version 8.0 (Boston, MA, USA) and Microsoft Excel 2024. Differences were considered statistically significant at *p < 0.05, **p < 0.01, and ***p < 0.001 compared to the control group.

## 3 Results

### 3.1 Anti‐Inflammatory Activity

#### 3.1.1 Protein Denaturation Assay

**FIGURE 1:**
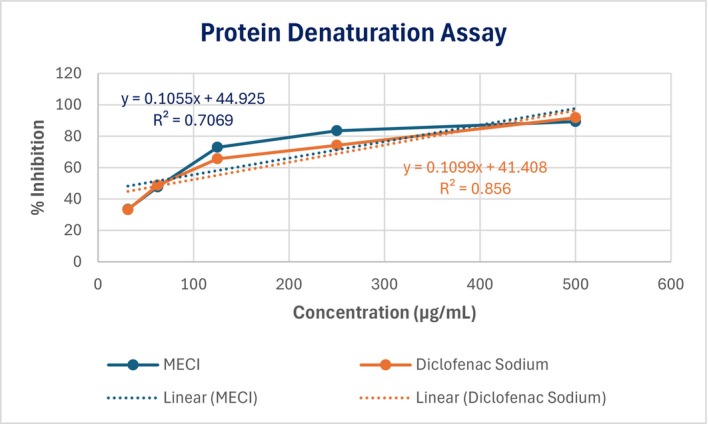
Evaluation of anti‐inflammatory activity of the methanolic extract of *C. infortunatum* through the protein denaturation assay. All values are expressed as mean ± SEM (*n* = 3). MECI = methanolic crude extract of *C. infortunatum*.

The anti‐inflammatory potential of the methanolic extract of *Clerodendrum infortunatum* (MECI) was evaluated using the protein denaturation assay. At a concentration of 500 μg/mL, MECI exhibited 89.29% inhibition of protein denaturation, comparable to the standard drug diclofenac, which showed 91.76% inhibition at the same concentration. The IC₅₀ value of MECI was determined to be 44.10, notably lower than that of diclofenac (78.18 μg/mL), indicating that the extract possesses potent anti‐inflammatory activity—possibly even greater than that of the reference drug under the tested conditions (Figure 1).

#### 3.1.2 Carrageenan‐Induced Paw Edema

**FIGURE 2:**
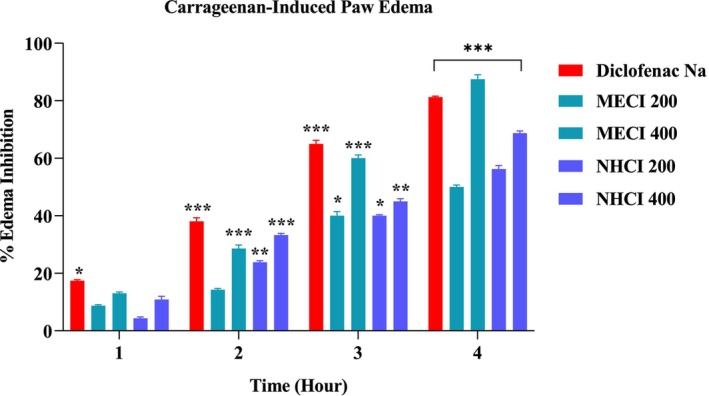
Evaluation of anti‐inflammatory activity of the methanolic extract and its *n*‐hexane fraction of *C. infortunatum* through the carrageenan‐induced paw edema test. Data are expressed as mean ± SEM. Statistical analysis was conducted using one‐way ANOVA, followed by Dunnett's multiple comparison test (*n* = 5). Significance levels were set at **p < 0.01, and ***p < 0.001 versus the control group.

In the present study, the anti‐inflammatory activity of the methanolic extract of *C. infortunatum* (MECI) and its n‐hexane fraction (NHF) was assessed using the carrageenan‐induced paw edema model in mice. The standard drug Diclofenac Na exhibited a modest anti‐edematous effect, with significant inhibition of paw edema beginning at the 2nd hour (*p* < 0.001) and peaking at 44 h. Similarly, MECI at 400 mg/kg demonstrated significant inhibition of paw edema from the 2nd hour onward, with sustained and pronounced effects at the 3rd hour and 24 h post‐carrageenan administration (*p* < 0.001). The n‐hexane fraction also showed a notable reduction in paw circumference, particularly after 24 h (Table 1). Across all tested groups—including paroxetine—the maximum anti‐inflammatory effect was observed at the 24‐h time point, indicating a time‐dependent and progressive inhibition of inflammation (Figure 2).

**TABLE 1:** Effects of the extract on the paw circumference of the treated mice.

Treatment group	Pre‐injection paw circumference (mm)	Postinjection paw circumference (mm)			
		1 h	2 h	3 h	4 h
Control	0.54 ± 0.66	0.46 ± 1.04	0.42 ± 0.33	0.40 ± 1.03	0.32 ± 1.14
Diclofenac Na	0.46 ± 0.44	0.38 ± 0.58*	0.26 ± 1.05***	0.14 ± 0.66***	0.06 ± 1.24***
MECI 200	0.52 ± 0.94	0.42 ± 0.33	0.36 ± 0.66	0.24 ± 1.03*	0.16 ± 1.44***
MECI 400	0.50 ± 0.33	0.40 ± 0.44	0.30 ± 1.14***	0.16 ± 1.12***	0.04 ± 2.02***
NHCI 200	0.50 ± 1.33*	0.44 ± 1.05	0.32 ± 0.94**	0.24 ± 0.24*	0.14 ± 0.44***
NHCI 400	0.48 ± 1.12	0.41 ± 0.88	0.28 ± 1.44***	0.22 ± 0.66**	0.10 ± 0.33***

*Note:* All values are expressed as Mean ± SEM. Statistical analysis was performed using one‐way analysis of variance (ANOVA), followed by Dunnett's multiple comparison test (*n* = 5). Abbreviations: MECI, methanolic crude extract of *C. infortunatum*; NHCI, *n*‐hexane fraction of *C. infortunatum*. *p < 0.05, **p < 0.01, and ***p < 0.001 compared with the control group.

### 3.2 Analgesic Activity

#### 3.2.1 Acetic Acid‐Induced Writhing Method

**FIGURE 3:**
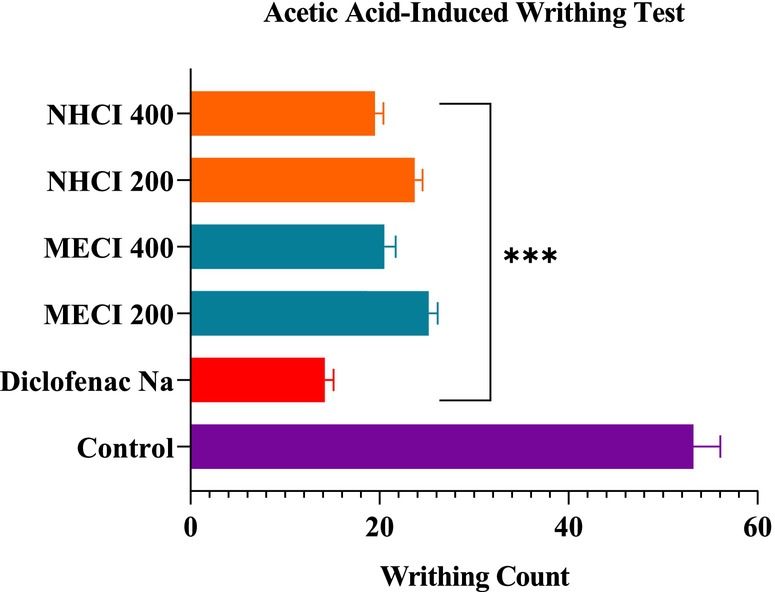
Evaluation of the analgesic activity of *C. infortunatum* through the acetic acid‐induced writhing method. Data are expressed as mean ± SEM. Statistical analysis was conducted using one‐way ANOVA, followed by Dunnett's multiple comparison test (*n* = 5). Significance levels were set at **p < 0.01, and ***p < 0.001 versus the control group.

The methanolic extract of *C. infortunatum* significantly reduced the number of writhes in a dose‐dependent manner, showing 52.63% and 61.47% inhibition at doses of 200 and 400 mg/kg body weight, respectively, compared to the untreated control group. This effect was comparable to that of the standard drug diclofenac sodium, which produced 73.30% inhibition (Table 2). Additionally, the n‐hexane soluble fraction, administered at 400 mg/kg body weight, also demonstrated notable antinociceptive activity, with 63.34% inhibition of writhing (Figure 3).

**TABLE 2:** Effects of *C. infortunatum* extract on the tested mice in the Acetic Acid‐Induced Writhing Method.

Animal group	Number of writhing	% of inhibition of writhing	% of writhing
Control	53.2 ± 2.84	0	100
Diclofenac Na	14.2 ± 0.92***	73.30	26.70
MECI 200	25.2 ± 0.93***	52.63	47.37
MECI 400	20.5 ± 1.21***	61.47	38.53
NHCI 200	23.7 ± 0.83***	55.45	44.55
NHCI 400	19.5 ± 0.90***	63.34	36.66

*Note:* All values are shown as Mean ± SEM, and statistical analysis is done using One‐Way Analysis of Variance (ANOVA). Subsequently, *n* = 5 is used for Dunnett's multiple‐comparison test, with *p < 0.05, **p < 0.01, and ***p < 0.001 compared with the control group. Abbreviations: MECI, Methanol crude extract of *C. infortunatum*; NHCI, *n*‐hexane fraction of *C. infortunatum*.

#### 3.2.2 Tail Immersion Test

**FIGURE 4:**
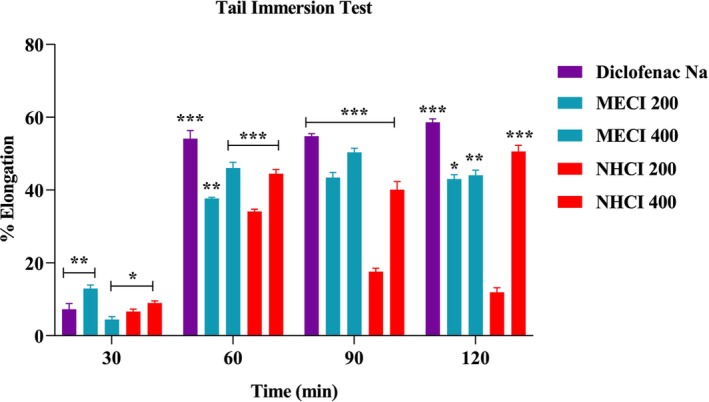
Evaluation of the analgesic activity of *C. infortunatum* through the tail immersion test. Data are expressed as mean ± SEM. Statistical analysis was conducted using one‐way ANOVA, followed by Dunnett's multiple comparison test (*n* = 5). Significance levels were set at **p < 0.01, and ***p < 0.001 versus the control group.

In the tail immersion test, the methanolic extract of *C. infortunatum* at doses of 200 and 400 mg/kg significantly increased reaction time at both 30 and 60 min (Table 3). The n‐hexane fraction, administered at 400 mg/kg, exhibited the most pronounced effect (*p* < 0.001) after 120 min. The percentage elongation of reaction time progressively increased over time (Figure 4). Similarly, the standard drug diclofenac sodium significantly prolonged response time (*p* < 0.001) at all tested time points.

**TABLE 3:** Effects of *C. infortunatum* extract on the tested mice in the Tail immersion test.

Treatment	Reaction time in seconds at time ± SEM				
	Pretreatment	30 min	60 min	90 min	120 min
Control	3.72 ± 0.31	4.78 ± 0.42	3.26 ± 0.24	3.60 ± 0.2	3.62 ± 0.2
Diclofenac Na	3.01 ± 0.11	5.08 ± 1.19**	7.10 ± 1.26***	7.96 ± 0.95***	8.74 ± 1.0***
MECI 200	3.12 ± 0.67	5.49 ± 0.72**	5.23 ± 1.20**	6.25 ± 0.37***	6.35 ± 0.5*
MECI 400	3.32 ± 0.16	5.00 ± 0.38*	6.04 ± 0.55***	7.25 ± 0.43***	6.47 ± 0.49**
NHCI 200	3.82 ± 0.28	5.12 ± 0.39*	4.95 ± 0.65***	4.37 ± 0.44***	4.11 ± 0.59
NHCI 400	3.42 ± 0.20	5.25 ± 0.99*	5.87 ± 0.43***	6.01 ± 0.40***	7.33 ± 0.59***

*Note:* All values are shown as Mean ± SEM, and statistical analysis is done using One‐Way Analysis of Variance (ANOVA). Subsequently, *n* = 5 is used for Dunnett's multiple‐comparison test, with *p < 0.05, **p < 0.01, and ***p < 0.001 compared with the control group. Abbreviations: MECI, methanol crude extract of *C. infortunatum*; NHCI, *n*‐hexane fraction of *C. infortunatum*.

### 3.3 Antidiarrheal Activity

#### 3.3.1 Castor Oil‐Induced Diarrhea

In the castor oil‐induced diarrheal model, the crude methanolic extract of *C. infortunatum* at 400 mg/kg exhibited a mild antidiarrheal effect in mice. In contrast, the n‐hexane fraction at the same dose (400 mg/kg) significantly reduced the frequency of diarrhea by 35.29% compared to the untreated control group. For reference, the standard drug loperamide demonstrated a stronger inhibitory effect, with a 45.09% reduction in diarrhea. These findings are summarized in Table 4.

**TABLE 4:** Effects of *C. infortunatum* extract on the tested mice in the castor oil‐induced diarrheal test.

Treatment	Total number of feces	% Inhibition of defecation	Total number of diarrheal feces	% Inhibition of diarrhea
Control	18.60 ± 0.33	—	10.20 ± 0.33	—
Loperamide	8.20 ± 0.33***	55.91	5.60 ± 0.33***	45.09
MECI 200	15.60 ± 0.33	16.12	8.00 ± 0.58	21.57
MECI 400	14.20 ± 0.88	23.65	7.40 ± 0.58*	27.45
NHCI 200	13.60 ± 0.58*	26.88	7.60 ± 0.33	25.49
NHCI 400	13.00 ± 0.58	30.1	6.60 ± 0.33	35.29

*Note:* All values are shown as Mean ± SEM, and statistical analysis is done using One‐Way Analysis of Variance (ANOVA). Subsequently, *n* = 5 is used for Dunnett's multiple‐comparison test, with *p < 0.05, **p < 0.01, and ***p < 0.001 compared with the control group. Abbreviations: MECI, Methanol crude extract of *C. infortunatum*; NHCI, *n*‐hexane fraction of *C. infortunatum*.

### 3.4 Antipyretic Activity

#### 3.4.1 Brewer's Yeast‐Induced Pyrexia

**FIGURE 5:**
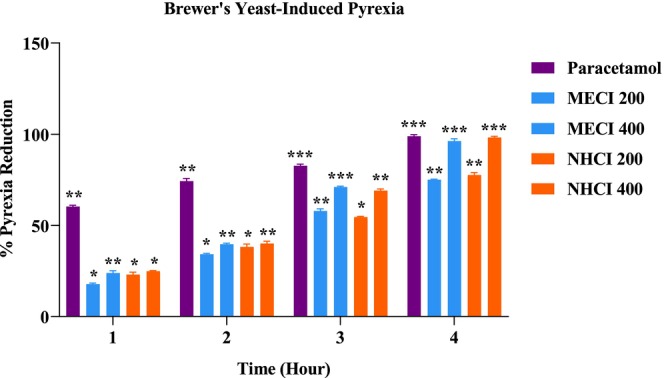
Evaluation of the antipyretic activity of *C. infortunatum* through the Brewer's yeast‐induced pyrexia method. Data are expressed as mean ± SEM. Statistical analysis was conducted using one‐way ANOVA, followed by Dunnett's multiple comparison test (*n* = 5). Significance levels were set at **p < 0.01, and ***p < 0.001 versus the control group.

The methanolic leaf extract of *C. infortunatum* and its n‐hexane fraction significantly reduced yeast‐induced hyperthermia in a dose‐dependent manner, with the effect remaining statistically significant for up to 4 h postadministration, as shown in Table 5. The greatest antipyretic activity was observed at the 400 mg/kg dose, where both the methanolic extract and the n‐hexane fraction achieved marked reductions in pyrexia—96.22% and 98.18%, respectively—as illustrated in Figure 5.

**TABLE 5:** Effect of *C. infortunatum* leaves extract on Brewer's yeast‐induced pyrexia in mice.

Group	Normal temperature	After 18 h of yeast administration	Rectal temperature after drug administration (°C)			
			1 h	2 h	3 h	4 h
Control	36.2 **±** 0.30	38.11 **±** 0.18	38.23 **±** 0.18	38.41 **±** 0.12	38.69 **±** 0.12	39 **±** 0.12
Paracetamol	36.23 **±** 0.28	38.22 **±** 0.07	37.02 **±** 0.13**	36.74 **±** 0.13**	36.58 **±** 0.15***	36.26 **±** 0.14***
MECI 200	36.3 **±** 0.22	38.02 **±** 0.14	37.72 **±** 0.15*	37.72 **±** 0.15*	37.04 **±** 0.13**	36.76 **±** 0.12**
MECI 400	36.34 **±** 0.34	38.11 **±** 0.13	37.69 **±** 0.12**	37.41 **±** 0.13**	36.86 **±** 0.12***	36.41 **±** 0.16***
NHCI 200	36.33 **±** 0.20	38.17 **±** 0.07	37.74 **±** 0.10*	37.47 **±** 0.09*	37.17 **±** 0.07*	36.74 **±** 0.14**
NHCI 400	36.31 **±** 0.28	38.14 **±** 0.18	37.69 **±** 0.20*	37.41 **±** 0.19**	36.88 **±** 0.18**	36.34 **±** 0.20***

*Note:* All values are shown as Mean ± SEM, and statistical analysis is done using One‐Way Analysis of Variance (ANOVA). Subsequently, *n* = 5 is used for Dunnett's multiple‐comparison test, with *p < 0.05, **p < 0.01, and ***p < 0.001 compared with the control group. Abbreviations: MECI, Methanol crude extract of *C. infortunatum*; NHCI, *n*‐hexane fraction of *C. infortunatum*.

### 3.5 In Silico Analysis

#### 3.5.1 ADME/T Analysis

The study confirms that all 15 compounds comply with Lipinski's Rule of Five, indicating their suitability for oral administration. Furthermore, their toxicological profiles were assessed using two online prediction tools—pKCSM (http://biosig.unimelb.edu.au/pkcsm/) and SwissADME (https://www.swissadme.ch/). The results suggest that most compounds are predicted to be nonmutagenic (AMES negative) and nonhepatotoxic, while a few (e.g., Hydrocinnamic acid derivative, 2‐Myristynoyl pantetheine) may exhibit hepatotoxicity, as summarized in Table 6. These predictions provide preliminary insights, and future in vivo tests are required.

**TABLE 6:** ADME/T analysis of the selected compounds of *C. infortunatum*.

Compounds name	Absorption		Distribution		Metabolism	Excretion	Toxicity		Drug likeliness	Bioavailability
	Water solubility (log mol/L)	Intestinal absorption (Human) (% Absorbed)	VDss (Human) (log L/kg)	BBB permeability (log BB)	CYP3A4 substrate	Total clearance (log mL/min/kg)	AMES toxicity	Hepatotoxicity		
Mannosamine	−1.694	26.9	−0.08	−0.936	No	0.735	No	No	Yes	0.55
L‐Gala‐l‐ido‐octose	−1.805	19.255	−0.08	−1.178	No	1.017	No	No	Yes	0.55
Ethyl isoallocholate	−4.734	97.702	−0.17	−0.796	Yes	0.748	No	No	Yes	0.55
Hydrocinnamic acid, o‐ [(1, 2, 3, 4‐tetrahydro‐2‐naphthyl) methyl] —	−3.989	96.477	−0.483	0.096	No	0.225	No	Yes	Yes	0.85
2,5‐Octadecadiynoic acid, methyl ester	−6.736	95.272	0.164	0.74	Yes	1.907	No	No	Yes	0.55
Octahydrochromen‐2‐one	−1.708	97.311	0.184	0.157	No	1.16	No	No	Yes	0.55
9‐Octadecenoic acid, (2‐phenyl‐1,3‐dioxolan‐4‐yl) methyl ester, cis—	−6.948	91.698	0.333	−0.551	Yes	1.501	No	No	Yes	0.55
Propanoic acid, 2‐(3‐acetoxy‐4,4,14‐trimethylandrost‐8‐en‐17‐yl)—	−3.607	99.835	−0.732	−0.149	Yes	0.29	No	No	Yes	0.85
10‐Heptadecen‐8‐ynoic acid, methyl ester, (E)—	−6.722	94.31	0.222	0.734	Yes	1.901	No	No	Yes	0.55
Hexadecanoic acid, methyl ester	−6.927	92.335	0.334	0.749	Yes	1.861	No	No	Yes	0.55
Methyl 9‐cis,11‐trans‐octadecadienoate	−7.343	92.66	0.272	0.767	Yes	2.028	No	No	Yes	0.55
9,12,15‐Octadecatrienoic acid, methyl ester, (Z, Z, Z)—	−7.232	93.166	0.246	0.757	Yes	2.086	No	No	Yes	0.55
Phytol	−7.535	90.643	0.385	0.793	Yes	1.686	No	No	Yes	0.55
Heptadecanoic acid, 16‐methyl‐, methyl ester	−7.522	91.793	0.272	0.786	Yes	1.775	No	No	Yes	0.55
2‐Myristynoyl pantetheine	−4.319	49.908	−0.967	−1.089	Yes	0.6	No	Yes	Yes	0.55

#### 3.5.2 Molecular Docking Study

Molecular docking was performed to evaluate the potential pharmacological activities of the identified compounds against four relevant target proteins (Table 7). For analgesic and anti‐inflammatory activities, the compounds were docked with the cyclooxygenase‐2 enzyme (PDB: 5IKR). Among the tested compounds, hydrocinnamic acid, o‐[(1,2,3,4‐tetrahydro‐2‐naphthyl) methyl]‐ showed the highest binding affinity with docking scores of −9.0 kcal/mol, outperforming the reference drug diclofenac sodium.

**TABLE 7:** Docking scores of the selected *C. infortunatum* compounds.

Compounds	PubChem CID	Docking score (Kcal/mol)		
		Anti‐inflammatory & analgesic (5IKR)	Antidiarrheal (5ZHP)	Antipyretic (4Y5K)
Mannosamine	440049	−5.4	−5.3	−4.4
L‐Gala‐l‐ido‐octose	219659	−5.3	−5.4	−5.1
Ethyl isoallocholate	6452096	−5	−8.7	**−5.6**
Hydrocinnamic acid, o‐[(1,2,3,4‐tetrahydro‐2‐naphthyl)methyl]—	582809	**−9**	**−10.2**	−5.5
2,5‐Octadecadiynoic acid, methyl ester	42151	−6.8	−6.4	−4
Octahydrochromen‐2‐one	20487	−6.8	−6.8	−4.7
9‐Octadecenoic acid, (2‐phenyl‐1,3‐dioxolan‐4‐yl) methyl ester, cis—	21160048	−7.1	−8.3	−4.1
Propanoic acid, 2‐(3‐acetoxy‐4,4,14‐trimethylandrost‐8‐en‐17‐yl)—	631957	−6.6	−9.1	−5.6
10‐Heptadecen‐8‐ynoic acid, methyl ester, (E)—	5367407	−6.3	−6.4	−4
Hexadecanoic acid, methyl ester	8181	−6.2	−6.5	−3.5
Methyl 9‐cis,11‐trans‐octadecadienoate	11748436	−6.9	−6.3	−3.9
9, 12, 15‐Octadecatrienoic acid, methyl ester, (Z, Z, Z)—	5319706	−6.8	−6.7	−4
Phytol	5280435	−6.5	−7.3	−4.3
Heptadecanoic acid, 16‐methyl‐, methyl ester	110444	−6.4	−6.6	−3.9
2‐Myristynoyl pantetheine	535560	−6.8	−7.9	−3.9
Diclofenac Na	3033	−8.1		
Loperamide	3955	—	−8.5	—
Paracetamol	1983	—	—	−4

For antidiarrheal activity, docking was carried out against the M_3_ muscarinic acetylcholine receptor (PDB: 5ZHP). The same hydrocinnamic acid derivative demonstrated the strongest interaction with a docking score of −10.2 kcal/mol, which was higher than that of the reference drug, loperamide.

**FIGURE 6:**
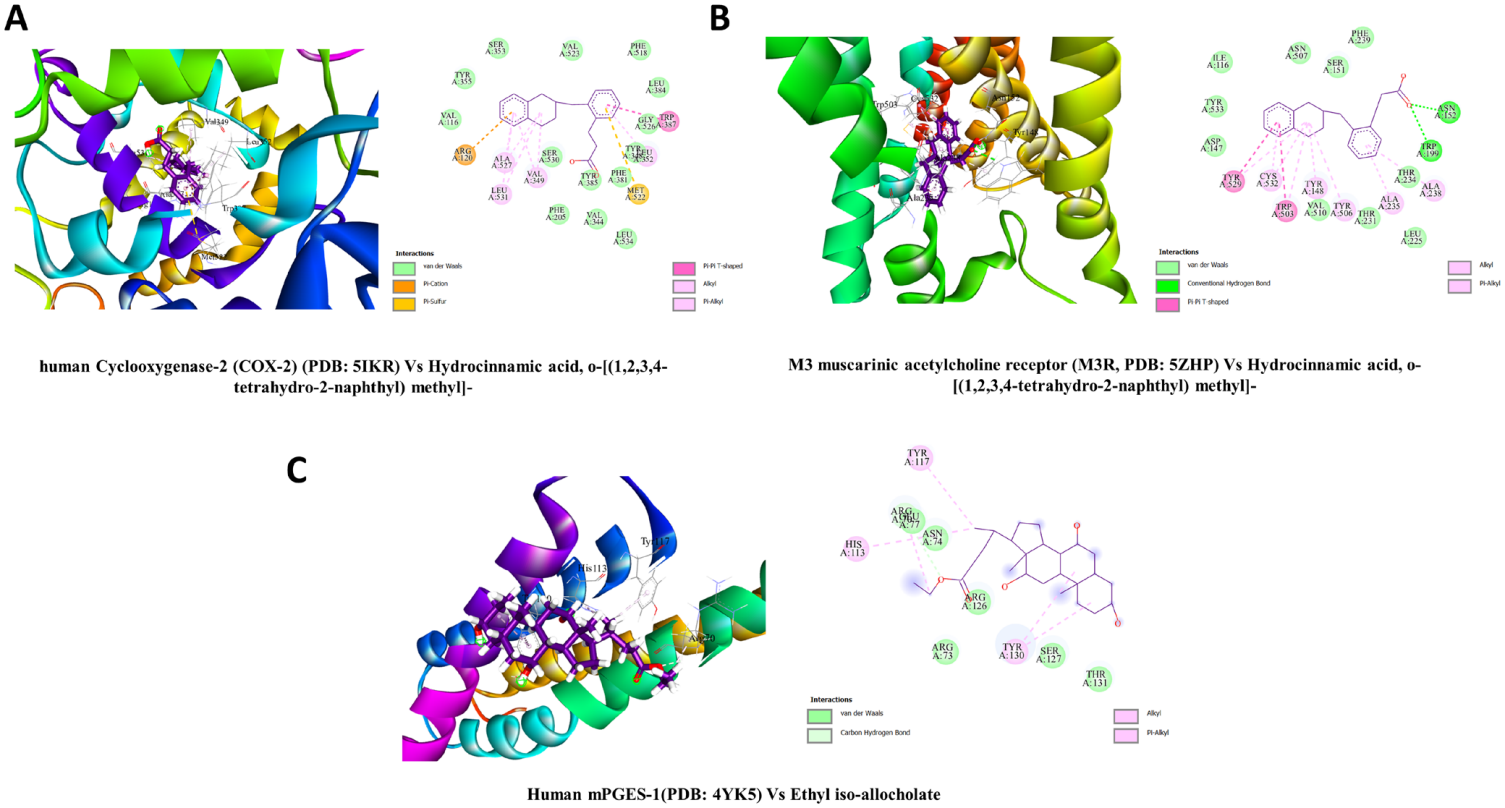
Molecular docking interactions of top docked compounds from the *C. infortunatum* leaves against important drug targets.

In the case of antipyretic activity, docking against microsomal prostaglandin E synthase‐1 (mPGES‐1) (PDB: 4YK5) revealed that ethyl isoallocholate exhibited the highest binding affinity (−5.6 kcal/mol). Detailed interaction profiles and docking scores are summarized in Table 8, while the binding interactions are illustrated in Figure 6.

**TABLE 8:** Binding affinity and nonbinding interactions of *C. infortunatum* compounds for anti‐inflammatory and analgesic (PDB: 5IKR), antidiarrheal (PDB: 5ZHP), and antipyretic (PDB: 4YK5) activity.

Compound	Receptor	Binding affinity (kcal/mol)	Bond type	Amino acids
Hydrocinnamic acid, o‐[(1,2,3,4‐tetrahydro‐2‐naphthyl)methyl]—	5IKR	−9	Pi‐Cation	ARG120
			Pi‐Sulfur	MET522
			Pi‐Pi T‐shaped	TRP387
			Alkyl	VAL349, ALA527, LEU531
			Pi‐Alkyl	LEU352, VAL349, ALA527, LEU531
	5ZHP	−10.2	Conventional Hydrogen Bond	ASN152, TRP199
			Pi‐Pi T‐shaped	TRP503, TYR529
			Alkyl	CYS532
			Pi‐Alkyl	TYR148, TRP503, TYR506, TYR529, ALA235, ALA238, CYS532
Ethyl isoallocholate	4YK5	−5.6	Carbon Hydrogen Bond	ARG70
			Alkyl	ARG70
			Pi‐Alkyl	HIS113, TYR117, TYR130

### 3.6 Frontier Molecular Orbitals (FMOs) Evaluations

**FIGURE 7:**
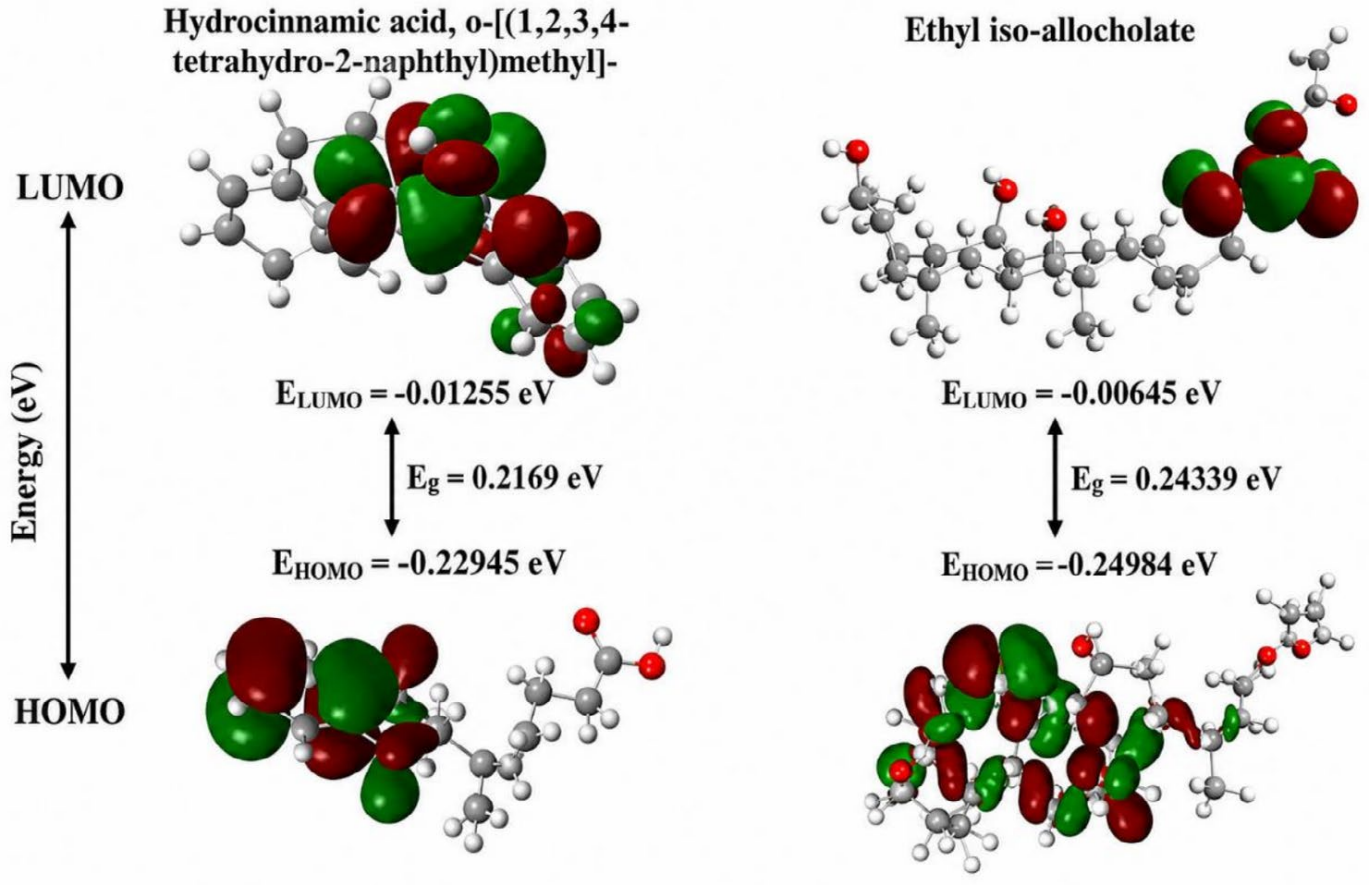
DFT‐based molecular orbital and reactivity descriptor profiling of selected compounds.

DFT analysis was performed on the two top‐ranking compounds from molecular docking: hydrocinnamic acid, o‐[(1,2,3,4‐tetrahydro‐2‐naphthyl) methyl]‐ and ethyl isoallocholate. The results revealed that hydrocinnamic acid exhibits a higher HOMO energy (−0.22945 au) and a lower LUMO energy (−0.01255 au) than ethyl isoallocholate, indicating enhanced electron‐donating and electron‐accepting capabilities, respectively. Notably, it possesses a smaller HOMO–LUMO energy gap (0.2169 au), higher global softness (8.26446 au^−1^), and greater electrophilicity index (0.067501 au)—all of which collectively point to superior chemical reactivity, favorable interaction potential with biological targets, and likely improved bioavailability. In contrast, ethyl isoallocholate displays a wider energy gap and lower reactivity descriptors, suggesting comparatively reduced reactivity (Table 9 and Figure 7).

**TABLE 9:** Molecular orbital and reactivity descriptor analysis.

Parameter	Hydrocinnamic acid, o‐[(1,2,3,4‐tetrahydro‐2‐naphthyl) methyl]‐	Ethyl isoallocholate
A = HOMO	−0.22945	−0.24984
I = LUMO	−0.01255	−0.00645
E Gap = (I‐A)	0.2169	0.24339
Chemical Potential (μ) = −(I + A)/2	0.121	0.128145
Hardness (ղ) = (I‐A)/2	0.10845	0.121695
Softness (σ) = 1/μ	8.26446	7.80366
Electronegativity (X) = (I + A)/2	−0.121	−0.128145
Electrophilicity (ω) = μ^2^/2ղ	0.067501	0.067468

### 3.7 PASS Prediction

PASS prediction analysis was used to screen 15 physiologically active chemicals for anti‐inflammatory, analgesic, antidiarrheal, and antipyretic effects. Table 10 displays the findings of this investigation.

**TABLE 10:** PASS prediction of biologically active compounds of *C. infortunatum* leaves.

Compound name	Biological activity							
	Anti‐inflammatory		Analgesic		Antidiarrheal		Antipyretic	
	Pa	Pi	Pa	Pi	Pa	Pi	Pa	Pi
Mannosamine	0.282	0.180	0.536	0.020	—	—	0.188	0.126
L‐Gala‐l‐ido‐octose	0.600	0.032	0.353	0.144	—	—	0.201	0.113
Ethyl isoallocholate	0.599	0.032	0.543	0.018	—	—	—	—
Hydrocinnamic acid, o‐ [(1, 2, 3, 4‐tetrahydro‐2‐naphthyl) methyl] —	0.360	0.036	0.332	0.158	0.187	0.116	0.485	0.015
2,5‐Octadecadiynoic acid, methyl ester	0.760	0.009	0.412	0.100	—	—	—	—
Octahydrochromen‐2‐one	0.280	0.181	0.458	0.064	—	—	0.260	0.065
9‐Octadecenoic acid, (2‐phenyl‐1,3‐dioxolan‐4‐yl) methyl ester, cis—	0.777	0.008	0.307	0.177	—	—	—	—
Propanoic acid, 2‐(3‐acetoxy‐4,4,14‐trimethylandrost‐8‐en‐17‐yl)—	0.850	0.005	0.652	0.005	—	—	—	—
10‐Heptadecen‐8‐ynoic acid, methyl ester, (E)—	0.580	0.036	0.461	0.062	—	—	0.177	0.139
Hexadecanoic acid, methyl ester	0.510	0.054	0.538	0.019	0.172	0.149	0.323	0.038
Methyl 9‐cis,11‐trans‐octadecadienoate	0.664	0.020	0.552	0.015	—	—	0.255	0.068
9,12,15‐Octadecatrienoic acid, methyl ester, (Z,Z,Z)—	0.803	0.006	0.571	0.011	—	—	0.218	0.098
Phytol	0.458	0.070	0.300	0.182	—	—	—	—
2‐Myristynoyl pantetheine	—	—	—	—	—	—	—	—
Heptadecanoic acid, 16‐methyl‐, methyl ester	0.392	0.100	0.490	0.042	—	—	0.253	0.070

## 4 Discussion

This study provides compelling evidence that the methanolic extract (MECI) and n‐hexane fraction (NHF) of *Clerodendrum infortunatum* leaves possess significant anti‐inflammatory, analgesic, antipyretic, and antidiarrheal properties. These findings align closely with the plant's longstanding use in Ayurvedic and folk medicine for managing inflammatory conditions, pain, fever, and gastrointestinal disturbances (Prashith Kekuda et al. 2019). The observed bioactivities likely stem from the synergistic interplay of a range of phytochemicals present in the extracts, including hydrocinnamic acid derivatives, fatty acid esters, phytosterols, and triterpenoids (Debnath et al. 2024).

The anti‐inflammatory potential of MECI was first evaluated using the protein denaturation assay, a model that mimics inflammatory tissue damage by unfolding native proteins and inducing autoantigen formation. MECI inhibited denaturation by 89.29%, nearly matching the efficacy of diclofenac sodium (91.76%) and exhibiting a lower IC₅₀ value (44.10 μg/mL), suggesting strong protective effects on protein integrity (Chandra et al. 2012). This activity corroborates previous reports on related *Clerodendrum* species, reinforcing the genus's relevance in traditional anti‐inflammatory remedies (Debnath et al. 2024).

Further validation came from the carrageenan‐induced paw edema model, a well‐established in vivo method for screening anti‐inflammatory agents. Both MECI and NHF significantly suppressed paw swelling in a dose‐dependent manner, with the highest dose (400 mg/kg) showing maximal inhibition after 24 h (*p* < 0.001). Carrageenan triggers a biphasic inflammatory response: an early phase (0–2 h) mediated by histamine and serotonin, and a delayed phase (3–5 h) driven by prostaglandins and bradykinin (Winter et al. 1962) (Mansouri et al. 2015). The pronounced effect during the later phase implies that the extracts likely interfere with prostaglandin synthesis, possibly via cyclooxygenase (COX) inhibition (Mansouri et al. 2015). Molecular docking results support this mechanism: a hydrocinnamic acid derivative, o‐[(1,2,3,4‐tetrahydro‐2‐naphthyl)methyl], showed high binding affinity for COX‐2 (−9.0 kcal/mol), forming stable interactions with key residues (SER353, MET522, TYR355), outperforming diclofenac (−8.1 kcal/mol). This underscores COX‐2 as a plausible molecular target, consistent with its central role in inflammation (Minghetti 2004).

Analgesic efficacy was confirmed through both peripheral and central pain models. In the acetic acid‐induced writhing test, MECI and NHF markedly reduced writhing episodes in a dose‐dependent fashion, with the highest inhibition (63.34%) approaching that of diclofenac (73.3%). This model reflects peripheral analgesia, primarily through suppression of prostaglandins E_2_ and F_2_α, which sensitize pain receptors. Additionally, the tail immersion test revealed a significant increase in response latency to thermal stimuli, indicating central nervous system‐mediated pain relief (Prashith Kekuda et al. 2019). The presence of both peripheral and central components of analgesia—evident from activity in both phases of the formalin test—points to a dual‐action mechanism. Notably, similar dual analgesic profiles have been reported for other species within the genus, including *Clerodendrum viscosum* and *Clerodendrum serratum*, reinforcing a consistent and potentially class‐specific pharmacological signature across Clerodendrum species (Debnath et al. 2024).

The antipyretic activity was evaluated in the brewer's yeast‐induced pyrexia model. Both extracts (400 mg/kg) effectively normalized elevated rectal temperatures by 96%–98% within 4 h. Yeast‐induced fever is mediated by hypothalamic PGE_2_ synthesis via COX and microsomal prostaglandin E synthase‐1 (mPGES‐1) activation (El‐Mahmoudy et al. 2018). The observed antipyretic effect likely arises from inhibition of this pathway (Chang and Meuillet 2011). *In silico* analysis reinforced this hypothesis: ethyl isoallocholate demonstrated strong binding to mPGES‐1 (−5.6 kcal/mol), interacting with critical residues ARG70 and TYR117, similar to the mechanism of paracetamol.

In the castor oil‐induced diarrhea model, MECI and NHF exhibited moderate but statistically significant antidiarrheal effects. At 400 mg/kg, NHF reduced diarrheal episodes by 35.29%, compared to 45.09% for loperamide. Castor oil is metabolized to ricinoleic acid, which stimulates prostaglandin release in the gut, enhancing secretion and motility (Kaur et al. 2014). The antidiarrheal action of the extracts may thus involve suppression of prostaglandin‐driven hypersecretion and/or modulation of cholinergic signaling. Docking studies revealed that hydrocinnamic acid derivatives bound strongly to the M_3_ muscarinic receptor (−10.2 kcal/mol), even more effectively than loperamide (−8.5 kcal/mol), suggesting a dual mechanism involving both prostaglandin inhibition and muscarinic receptor antagonism (Fukushima et al. 2012) (Kruse et al. 2012).

Frontier Molecular Orbital (FMO) analysis—centered on the Highest Occupied Molecular Orbital (HOMO) and Lowest Unoccupied Molecular Orbital (LUMO)—offers fundamental insights into a molecule's electronic structure, reactivity, and interaction potential (Teles Fujishima et al. 2018). A narrow HOMO–LUMO energy gap generally signifies enhanced chemical reactivity, reflecting greater ease of electron donation (from HOMO) and acceptance (into LUMO). Moreover, the spatial orientation of these orbitals helps identify susceptible regions—particularly within π‐conjugated systems—for electrophilic, nucleophilic, or radical attacks, thereby supporting the prediction of binding hotspots and reaction pathways in drug–target or antioxidant processes (Tumilaar et al. 2025). In this DFT‐based study, hydrocinnamic acid, o‐ [(1,2,3,4‐tetrahydro‐2‐naphthyl)methyl]‐ exhibits a favorable electronic profile, suggesting improved bioavailability, whereas ethyl isoallocholate displays a distinct orbital configuration indicative of specialized electron‐transfer capability—potentially advantageous for redox‐active applications. Collectively, such electronic structure analyzes serve as a powerful foundation for rational drug design and targeted molecular optimisation in pharmaceutical research.

Pharmacokinetic profiling via ADME/T analysis showed that all major constituents comply with Lipinski's Rule of Five, indicating good oral bioavailability. Hydrocinnamic acid derivatives and ethyl isoallocholate exhibited high intestinal absorption (> 95%) and showed no signs of hepatotoxicity or mutagenicity in the Ames test. These findings align with prior studies on structurally similar compounds, affirming their drug‐like properties and safety.

Collectively, the observed pharmacological effects appear to arise from multi‐compound, multi‐target interactions, a hallmark of many traditional herbal medicines. In vivo findings support the involvement of both peripheral and central mechanisms of action, while in silico analyzes pinpoint COX‐2, microsomal prostaglandin E synthase‐1 (mPGES‐1), and the M_3_ muscarinic receptor as key molecular targets—proteins critically implicated in inflammation, pain, fever, and gastrointestinal motility regulation. This polypharmacological profile may confer therapeutic advantages over single‐target synthetic drugs by simultaneously modulating interconnected pathophysiological networks, thereby addressing the multifactorial nature of complex diseases with potentially greater efficacy and reduced risk of compensatory resistance mechanisms (Wang and Yang 2022). In summary, this study provides scientific validation for the ethnomedicinal uses of *C. infortunatum* and identifies hydrocinnamic acid derivatives and ethyl isoallocholate as lead compounds exhibiting strong binding affinity toward relevant targets and favorable pharmacokinetic properties. These findings not only substantiate traditional knowledge but also position *C. infortunatum* as a promising source of novel, multi‐target therapeutic agents. Future work should prioritize bioassay‐guided fractionation, detailed receptor‐binding and functional assays, and comprehensive preclinical safety and toxicity studies to facilitate the development of standardized, clinically viable phytomedicines.

Molecular docking was performed under the standard assumption that proteins remain rigid while ligands are flexible—a widely adopted approach that balances computational efficiency with reasonable accuracy for initial screening. However, we acknowledge that this simplification represents a limitation, as it does not account for induced‐fit conformational changes in the protein upon ligand binding, which can influence binding affinity and pose prediction. Future studies employing ensemble docking or molecular dynamics simulations would provide a more dynamic and realistic representation of ligand–protein interactions.

The combined in vivo and in silico findings suggest that multiple phytoconstituents may contribute to the observed pharmacological activities through interactions with several biological targets. Such polypharmacological behavior may enhance therapeutic efficacy by simultaneously modulating interconnected pathological pathways. However, multi‐target interactions may also increase the risk of unintended off‐target effects, adverse reactions, or complex pharmacodynamic interactions. Therefore, further mechanistic investigations, pharmacokinetic studies, and safety assessments are required to optimize the therapeutic potential of these bioactive compounds and establish their overall benefit–risk profile.

## 5 Conclusion

This study provides strong evidence that the methanolic extract (MECI) and the n‐hexane fraction (NHF) of *Clerodendrum infortunatum* exhibit significant anti‐inflammatory, analgesic, antipyretic, and antidiarrheal activities, as validated across multiple experimental models. These effects are mediated via both peripheral and central mechanisms, likely through inhibition of prostaglandin synthesis and modulation of key targets—COX‐2, mPGES‐1, and the M3 muscarinic receptor. Molecular docking confirmed strong binding of bioactive compounds (e.g., hydrocinnamic acid derivatives and ethyl isoallocholate) to these targets. ADME/T and PASS predictions further support their drug‐like properties, low toxicity, and high oral bioavailability. The findings substantiate the traditional use of *C. infortunatum* and highlight its phytoconstituents as promising leads for the development of natural therapeutics. Future work should focus on compound isolation, detailed mechanistic studies, and preclinical development for phytopharmaceutical applications.

## 6 Limitations of the Study

Despite the promising pharmacological findings, several limitations should be acknowledged. First, the in vivo experiments used a relatively small sample size (*n* = 5 per group), which may limit the statistical power and generalizability of the results. Second, the study evaluated the biological activities using crude methanolic extract and its n‐hexane fraction rather than isolated pure compounds; therefore, the exact phytochemicals responsible for the observed effects cannot be definitively identified. Third, although molecular docking, ADME/T analysis, PASS prediction, and DFT calculations provided valuable mechanistic insights, these in silico results require further validation through detailed biochemical and molecular assays. In addition, the pharmacological investigations were performed only in animal models; thus, the findings may not directly translate into human physiological responses. Additionally, only male Swiss albino mice were included in the present study. Sex‐dependent physiological and pharmacological differences may influence treatment responses; therefore, future studies should include both male and female animals to improve the generalizability of the findings. Furthermore, although no overt signs of toxicity were observed during the experimental period, comprehensive toxicological evaluations, including subacute, chronic, reproductive, and organ‐specific toxicity studies, were not performed. The absence of these investigations represents a limitation for the clinical translation and long‐term safety assessment of the extracts and their bioactive constituents. Finally, long‐term toxicity, pharmacokinetic behavior, and detailed receptor‐binding studies were not explored in the present work. These limitations may affect the strength of the conclusions and underscore the need for further studies on compound isolation, mechanistic investigations, and comprehensive preclinical and clinical evaluations to confirm the therapeutic potential of *Clerodendrum infortunatum*. Future studies will focus on the bioassay‐guided fractionation and isolation of the identified lead compounds from *Clerodendrum infortunatum*, followed by structural characterization and experimental validation of their pharmacological activities.

## Author Contributions

**Mohi Uddin:** writing – original draft, project administration, writing – review and editing, methodology, supervision, investigation, data curation, software. **Sabikun Naher:** data curation, formal analysis, writing – original draft, writing – review and editing, methodology, software. **Md. Hossain Rasel:** data curation, writing – review and editing, methodology, software, writing – original draft. **Md. Jahirul Islam Mamun:** data curation, methodology, software, writing – review and editing, writing – original draft. **Md. Nazmul Hasan:** data curation, methodology, writing – review and editing, software, writing – original draft. **Nazmul Hasan Eshaque:** writing – original draft, data curation, methodology, software, writing – review and editing. **Md. Mahmudul Hasan:** writing – original draft, methodology, validation, formal analysis.

## Funding

The authors have nothing to report.

## Conflicts of Interest

The authors declare no conflicts of interest.

## Data Availability

The datasets generated and analyzed during the current study are available from the corresponding author upon reasonable request. To promote transparency and reproducibility, the authors will consider depositing relevant datasets, including molecular docking outputs and raw experimental data, in an appropriate public repository in future studies.
